# Reduction of Proteinuria in a Patient With Primary Aldosteronism by Angiotensin II Receptor Blocker Administration

**DOI:** 10.1210/jcemcr/luac021

**Published:** 2022-11-30

**Authors:** Junjiro Rikitake, Kenji Ashida, Mami Miura, Masatoshi Nomura

**Affiliations:** Division of Endocrinology and Metabolism, Department of Internal Medicine, Kurume University School of Medicine, Kurume, Fukuoka 830-0011, Japan; Division of Endocrinology and Metabolism, Department of Internal Medicine, Kurume University School of Medicine, Kurume, Fukuoka 830-0011, Japan; Division of Endocrinology and Metabolism, Department of Internal Medicine, Kurume University School of Medicine, Kurume, Fukuoka 830-0011, Japan; Division of Endocrinology and Metabolism, Department of Internal Medicine, Kurume University School of Medicine, Kurume, Fukuoka 830-0011, Japan

**Keywords:** aldosterone, angiotensin II, mineralocorticoid receptor antagonists, proteinuria, renin-angiotensin aldosterone system

## Abstract

The renin–angiotensin–aldosterone system (RAAS) is a major target for treating hypertension and preventing various complications. Mineralocorticoid receptor (MR) antagonists are recommended as specific drugs to ameliorate hyperactive MR signaling, especially for patients with idiopathic hyperaldosteronism. However, the clinical implications of an increased RAAS activity and angiotensin II level induced by MR antagonist administration remain unclear. A 72-year-old Japanese man was referred to our university hospital for refractory hypertension management. He has also had type 2 diabetes mellitus and nephropathy for 8 years. MR antagonists, initiated based on the diagnosis of primary aldosteronism, effectively improved his hypertension. However, proteinuria of 2.5 g/g creatinine, concomitant with an increase in both active renin concentration and plasma aldosterone concentration, occurred. Additional administration of an angiotensin II receptor blocker successfully reduced the plasma aldosterone concentration and proteinuria (<0.3 g/g creatinine). Preserved renal function was confirmed for 1 year thereafter. In conclusion, this case suggests that the angiotensin II receptor is a potential target to treat proteinuria concomitant with primary aldosteronism. RAAS reactivation should be considered when an MR antagonist is initiated for patients with primary aldosteronism, especially idiopathic hyperaldosteronism.

Regulation of the renin–angiotensin–aldosterone system (RAAS) is the mainstream treatment of hypertension and prevention of hypertension-related complications. Hyperactivity of mineralocorticoid receptor (MR) signals leads to disorders of the cardiovascular system and kidneys via chronic inflammation and fibrosis in the related organs [1]. Administration of angiotensin II receptor blockers (ARBs) and angiotensin-converting enzyme (ACE) inhibitors has been reported to be effective in preventing cardiovascular events and renal dysfunction through RAAS regulation [2].

Primary aldosteronism (PA) frequently occurs with hypertension, accounting for approximately 5% to 13% of all hypertension cases [3]. Hypertension and hyperaldosteronism are associated with a high risk of dysfunction in various organs, including renal and cardiovascular diseases. Notably, patients with PA have a higher risk of developing chronic kidney disease (CKD) than those with essential hypertension, independent of their blood pressure levels. MR antagonist (MRA) administration in patients with PA is effective in preventing cardiovascular disorders, although the effect is achieved more slowly than the effects of surgical removal of aldosterone-producing tumors [3]. Proper interventions to reduce hyperactivated MR-related signaling are required from the early stages of disease to avoid the progression of systemic complications.

The blockade of MR signaling using MRAs is a specific and effective medical treatment and is recommended especially for idiopathic hyperaldosteronism [3]. Additionally, elevation in plasma renin activity (PRA) by more than 1 ng/mL/hour, is a potential indicator of MRA administration efficacy [4]. However, issues related to reactivation of the RAAS [5] and increased angiotensin II levels by MRA administration remain unresolved.

Herein, we present a patient with idiopathic hyperaldosteronism concomitant with diabetes mellitus and CKD with macroproteinuria. Diagnosis of PA was considered a key factor in preventing CKD progression in this case. This case provides evidence that angiotensin II receptor blockade is a potential therapeutic strategy for patients with PA to prevent the progression of systemic complications.

## Case Presentation

A 72-year-old Japanese man with hypertension was admitted to our university hospital for examination and treatment of PA. He had undergone a subtotal thyroidectomy due to Graves' disease at 26 years of age and continued levothyroxine to manage hypothyroidism. He had a history of hemorrhage in the brainstem at 54 years of age, when he was diagnosed with hypertension, and had been taking antihypertensive drugs since then. Sustained hypertension despite various drug regimens and a relatively high ratio of plasma aldosterone concentration (PAC) (209 pg/mL [579 pmol/L]; reference range [RR], 30-160 pg/mL [83-443 pmol/L]; by radioimmunoassay) to PRA (0.7 μg/L/hour; RR, 0.2-2.3; by radioimmunoassay) in July 2018 led to further evaluation for PA at 69 years of age in November 2018. Notably, his urinalysis did not reveal proteinuria following administration of 100 mg/day irbesartan, 50 mg/day eplerenone, and 10 mg/day amlodipine. At the same time, his blood pressure was 135/73 mmHg.

## Diagnostic Assessment

**Figure 1.**
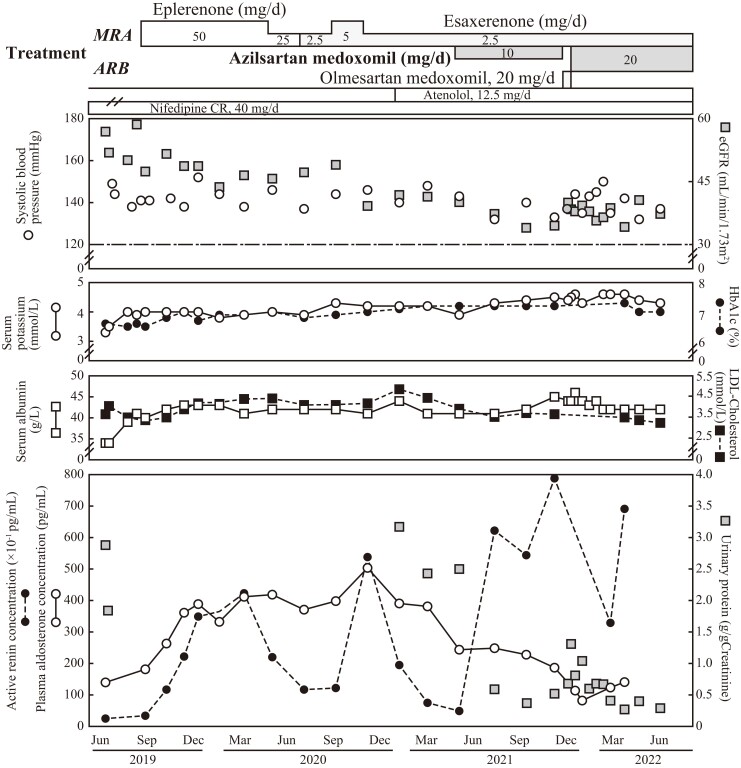
Clinical course of the present case. Discontinuation of angiotensin II receptor blocker (ARB) and mineralocorticoid receptor antagonist (MRA) led to an exacerbation of proteinuria, probably due to diabetes-related nephropathy. Administration of eplerenone or esaxerenone increased both active renin concentration (closed circles with dashed lines, bottom panel) (ARC) and plasma aldosterone concentration (open circles with solid lines, bottom panel) (PAC). On the contrary, addition of atenolol decreased both ARC and PAC. However, the co-administration of azilsartan decreased proteinuria (gray squares, bottom panel) along with a further decrease in PAC, although MRA and atenolol based on nifedipine administration did not reduce proteinuria. Increased serum albumin levels (open squares with solid lines) correlated with decreased blood pressure and azilsartan co-administration, although single use of eplerenone or esaxerenone did not induce this effect. Low-density lipoprotein cholesterol levels (closed squares with dashed lines) did not decrease during single MRA administration (third upper panel). Furthermore, the estimated glomerular filtration rate (eGFR) (gray squares, first upper panel) decreased gradually even after MRA (eplerenone or esaxerenone) administration; however, it was preserved following 10 mg/day azilsartan administration. Systolic blood pressure (open circles, first upper panel) gradually decreased below 140 mmHg. Moreover, serum potassium levels (open circle with lines, second upper panel) were preserved within reference range and HbA1c levels (closed circles with dashed lines, second upper panel) were maintained around 7% during the study. Abbreviations: ARB, angiotensin II receptor blocker; ARC, active renin concentration; eGFR, estimated glomerular filtration rate; HbA1c, hemoglobin A1c; MRA, mineralocorticoid receptor antagonist; PAC, plasma aldosterone concentration.

**Figure 2.**
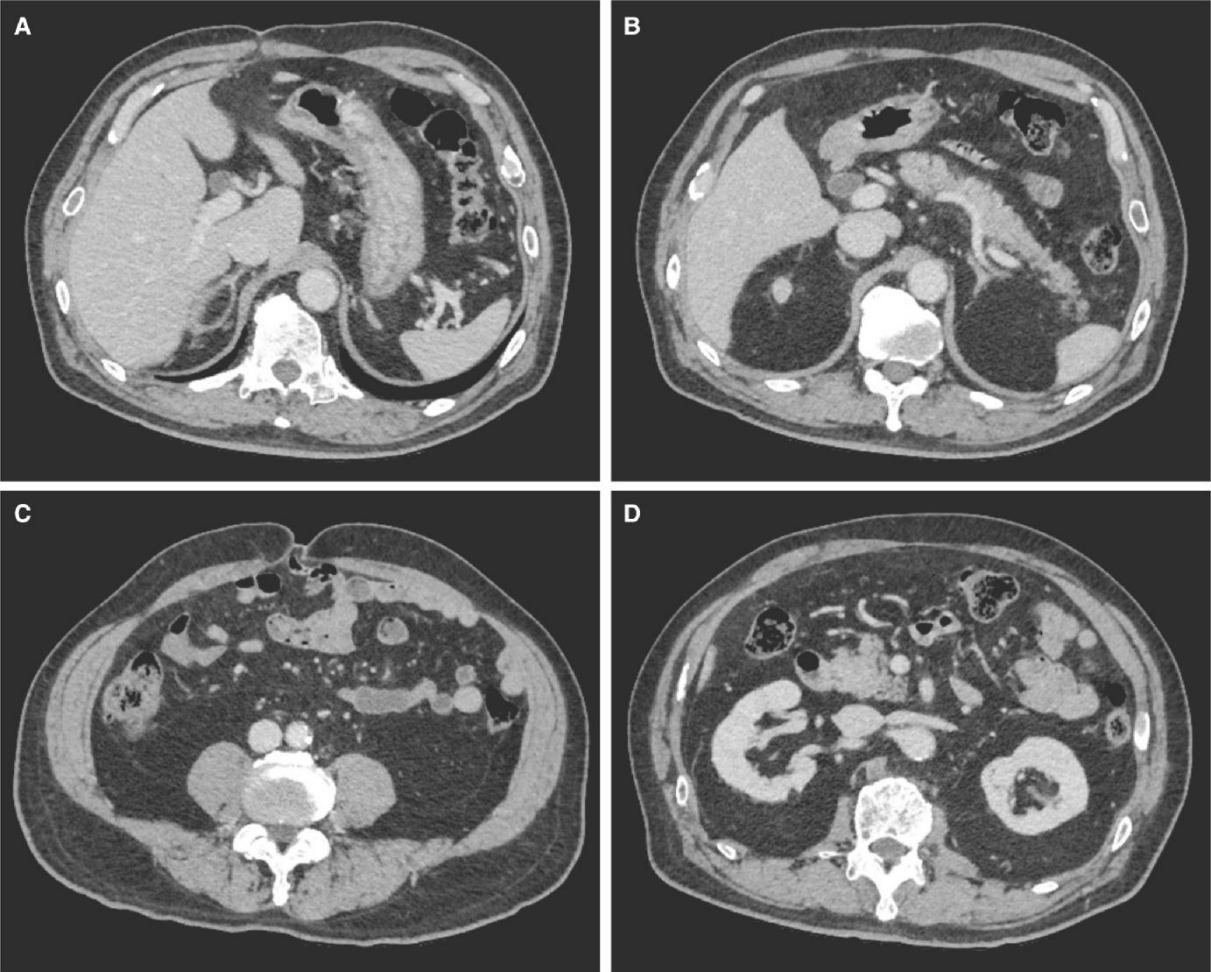
Computed tomography examination. No definite adrenal nodule was found in the bilateral adrenal glands. (A) Right adrenal gland. (B) Left adrenal gland. (C) Visceral adipose tissue accumulation was noted compared with the subcutaneous adipose tissue. High accumulation of visceral fat (145.4 cm^2^) and a visceral/subcutaneous adipose tissue ratio of 0.53 (reference range: <0.4) were observed. (D) Thinned bilateral cortexes of kidneys are presented.

The patient's laboratory examinations, performed in the absence of antihypertensive drugs except for calcium antagonists (40 mg/day of nifedipine CR) administered for over 2 months, revealed hypokalemia (3.3 mmol/L; RR, 3.6-4.8 mmol/L). Urinalysis demonstrated proteinuria; however, no occult blood was observed (Fig. 1). The captopril challenge test revealed defects in the suppression of both PAC and PAC/PRA (Table 1) [6]. The furosemide upright test revealed a suppressed PRA of < 2 μg/L/hour (maximum PRA of 1.3 μg/L/hour) (Table 1) [7]. The saline infusion test using 2 L saline over 4 hours did not suppress PAC (Table 1) [6]. However, no definitive nodule was found in the adrenal glands (Fig. 2A and 2B). Visceral adipose accumulation was observed with a high visceral fat area and visceral/subcutaneous adipose tissue ratio (Fig. 2C). Upon drip infusion of adrenocorticotropic hormone, adrenal venous sampling indicated neither step-ups nor laterality of PAC in the adrenal veins (Table 2). Regarding serum cortisol, the circadian rhythm was preserved, and autonomous cortisol production was ruled out based on the normal 1-mg dexamethasone suppression test.

**Table 1.** Confirmational examination of primary aldosteronism

Time (min)	0	60	90	120	240
Captopril challenge test					
PRA, μg/L/hour	0.4	0.4	0.4	0.4	NA
PAC, pg/mL [pmol/L]	136.6 [379]	107.3 [298]	86.0 [239]	101.3 [281]	NA
ARR	342	268	215	253	NA
Furosemide upright test					
PRA, μg/L/hour	0.9	NA	NA	1.3	NA
PAC, pg/mL [pmol/L]	165.8 [460]	NA	NA	310.8 [862]	NA
Saline infusion test					
PRA, μg/L/hour	4.9	NA	NA	NA	2.0
PAC, pg/mL [pmol/L]	228.2 [633]	NA	NA	NA	185.4 [514]

Abbreviations: ARR, aldosterone/renin ratio; NA, not applicable; PAC, plasma aldosterone concentration; PRA, plasma renin activity.

**Table 2.** Results of adrenal venous sampling examination

Parameters	PAC, pg/mL [pmol/L]	Cortisol, μg/dL [nmol/L]	PAC/Cortisol ratio
Sampling site			
Right adrenal vein	1802.6 [5000]	347.0 [9574]	5.2
Left adrenal vein	1479.1 [4103]	537.0 [14 816]	2.8
Inferior vena cava (proximal)	332.9 [923]	29.60 [817]	11.2
Inferior vena cava (distal)	378.5 [1050]	30.5 [841]	12.4
Right renal vein	325.7 [903]	25.80 [712]	12.6
Left renal vein (proximal)	1462.1 [4056]	80.20 [2213]	18.2
Left renal vein (distal)	350.7 [973]	26.40 [728]	13.3

Adrenal venous sampling was performed using a drip infusion of adrenocortical stimulating hormone (50 μg/hour).

Abbreviation: PAC, plasma aldosterone concentration.

## Treatment

Diabetic kidney disease (DKD) stage III was diagnosed based on proteinuria of 2.9 g/day and an estimated glomerular filtration rate (eGFR) of 56.9 mL/min/1.73 m^2^ (Fig. 1); bilateral renal cortical thinning was also present (Fig. 2D). The patient had been continuously treated for diabetes mellitus (along with moderate nonproliferative diabetic retinopathy) with 50 mg/day sitagliptin and 7.5 mg/day pioglitazone before and during this study period. Additionally, his hemoglobin A1c level was 6.6% (National Glycohemoglobin Standardization Program) and was maintained at approximately 7% during his clinical course (Fig. 1). Based on the diagnosis of idiopathic hyperaldosteronism with DKD, treatment with MRA was initiated along with administration of Ca-antagonists and management of salt intake at approximately 7 g/day. He had been treated with 50 mg/day eplerenone since September 2019; the dose was reduced to 25 mg/day in May 2020 due to fatigue and hypotension at home (<110 mmHg), although his blood pressure at hospital did not drop significantly. The MRA treatment was switched to 5 mg/day esaxerenone in July 2020 and reduced to 2.5 mg/day in November 2020 because his PRA or active renin concentration (ARC) was elevated. He had been taking 12.5 mg/day atenolol orally since January 2021 to manage hypertension; this decreased his ARC and PAC but could not mitigate proteinuria sufficiently. The addition of oral azilsartan 10 mg/day from May 2021 led to a continuous improvement in his urinary protein values from 3.2 to 0.3 g/g creatinine, and his ARC and PAC/ARC values increased and decreased, respectively (Fig. 1). His serum albumin level increased after hypertension management and was preserved at sufficiently high levels following MRA and ARB co-administration compared with the level during individual administration of MRAs. Additionally, low-density lipoprotein cholesterol levels decreased gradually following MRA and ARB co-administration (Fig. 1). The change from 10 mg/day azilsartan to 20 mg/day olmesartan led to a transient increase in proteinuria, which was reduced when azilsartan administration was restored at 20 mg/day.

## Outcome and Follow-Up

The blood pressure of the patient decreased gradually and has been maintained around the target range by administering multiple antihypertensive medications. Additionally, there has been no further progression of renal dysfunction (eGFR approximately 40 mL/min/1.73 m^2^ for over 1 year after a transient decrease). His potassium level has been regularly monitored and maintained within the normal range throughout the clinical course (Fig. 1).

## Discussion

RAAS reactivation associated with MRA administration is a potential risk factor for proteinuria progression, which may cause renal dysfunction and cardiovascular disease. MRA administration overrides PRA suppression via MR blockade, even in patients with PA, and has been suggested to elevate angiotensin and aldosterone sequentially [4, 5]. We confirmed PA using the 3 major tests [6-8] and ruled out hyperkalemia-induced hyperaldosteronism [8]. The present study revealed that clinicians should be aware of RAAS reactivation when using MRAs for treating patients with PA, especially idiopathic hyperaldosteronism, to achieve effective patient outcomes.

Regulation of reactivated RAAS using an ARB can reduce proteinuria and halt the progression of renal dysfunction in patients with PA. We first demonstrated a reduction in proteinuria following ARB co-administration in patients with PA treated with an MRA. This case also revealed that ARB administration reduced PAC, which had earlier increased, and MRA administration released PRA suppression. Both angiotensin II and aldosterone can exacerbate proteinuria and renal dysfunction independently [2], although reduction in eGFR might be associated with decreased proteinuria. Additive administration of an MRA can effectively reduce proteinuria and albuminuria in patients with CKD, including DKD [9]. Although sufficient MRA administration can reduce urinary albumin excretion by 50% [4], we observed a notable improvement in urinary albumin excretion in our case. Thus, based on our findings, we infer that suppressing angiotensin II activity may be important even after the MR blockade. In this context, total regulation of the RAAS, that is, the dual suppression of angiotensin II and aldosterone hyperactivity would be required to achieve the desired outcomes of PA treatment.

The RAAS activity, which includes aldosterone, should be regulated as a critical target for improving the prognosis of patients with MR-associated hypertension. RAAS regulation using ARB/ACE inhibitors is the gold standard treatment for hypertension, whereas hyperactivated MR signaling is a proposed critical target for post-RAAS regulation therapy [10]. Hyperactivated MR signals evoke inflammation and fibrosis, resulting in local remodeling and, subsequently, renal and cardiovascular dysfunction [1]. Obesity and diabetes mellitus are associated with a risk of hyperactivation of MR signaling [10]. The findings of the present study suggest that the regulation of hyperactivated RAAS activity using an ARB may be required for treating hypertension, even in conditions where PRA or ARC levels are suppressed, including PA. Additionally, each ARB drug may have varying efficacy in reducing proteinuria. Clinicians should be aware of the risk of exacerbated proteinuria and renal dysfunction due to MRA and ARB withdrawal, even during the washout period when conducting confirmational tests.

This case report has 4 limitations. First, further large-scale prospective studies are required to confirm our results. Second, we could not rule out the effect of reduced blood pressure and/or β-blocker administration on proteinuria or renal function, although the effectiveness of ARB administration in a patient with PA itself is a notable finding. Third, owing to the lack of data on proteinuria during single administration of MRAs, we could not investigate the effectiveness of high-dose MRAs completely. Fourth, we could not conclude whether we can apply this result to patients with aldosterone-producing adenomas.

## Learning Points

- We present the first case that demonstrated angiotensin II receptor as a potential target for treating proteinuria in a patient with PA.
- The blockade of RAAS reactivation associated with MRA administration was required to prevent proteinuria progression. Furthermore, prevention of renal dysfunction was observed for over 1 year.
- RAAS activity, including the activity of both aldosterone and angiotensin II, should be emphasized as a critical target for improving the prognosis of patients with MR-associated hypertension, including PA.

## Data Availability

Some or all datasets generated and/or analyzed during the current study are not publicly available but are available from the corresponding author on reasonable request.

## Abbreviations

ACE: angiotensin-converting enzyme
ARB: angiotensin II receptor blocker
ARC: active renin concentration
CKD: chronic kidney disease
DKD: diabetic kidney disease
eGFR: estimated glomerular filtration rate
MR: mineralocorticoid receptor
MRA: mineralocorticoid receptor antagonist
PA: primary aldosteronism
PAC: plasma aldosterone concentration
PRA: plasma renin activity
RAAS: renin–angiotensin–aldosterone system
